# Effect of a high protein diet and/or resistance exercise on the preservation of fat free mass during weight loss in overweight and obese older adults: a randomized controlled trial

**DOI:** 10.1186/s12937-017-0229-6

**Published:** 2017-02-06

**Authors:** Amely M. Verreijen, Mariëlle F. Engberink, Robert G. Memelink, Suzanne E. van der Plas, Marjolein Visser, Peter J.M. Weijs

**Affiliations:** 1 Department of Nutrition and Dietetics, Faculty of Sports and Nutrition, Amsterdam University of Applied Sciences, Dr. Meurerlaan 8, 1067 SM Amsterdam, Netherlands; 2 Department of Health Sciences, Faculty of Earth and Life Sciences, VU University Amsterdam, De Boelenlaan 1085, 1081 HV Amsterdam, Netherlands; 3 Department of Nutrition and Dietetics, Internal Medicine, VU University Medical Center, De Boelenlaan 1117, 1081 HV Amsterdam, Netherlands

**Keywords:** Older adults, Obesity, Weight loss, Fat free mass, High protein diet, Resistance exercise

## Abstract

**Background:**

Intentional weight loss in obese older adults is a risk factor for accelerated muscle mass loss. We investigated whether a high protein diet and/or resistance exercise preserves fat free mass (FFM) during weight loss in overweight and obese older adults.

**Methods:**

We included 100 overweight and obese adults (55–80 year) in a randomized controlled trial (RCT) with a 2 × 2 factorial design and intention-to-treat analysis. During a 10-week weight loss program all subjects followed a hypocaloric diet. Subjects were randomly allocated to either a high protein (1.3 g/kg body weight) or normal protein diet (0.8 g/kg), with or without a resistance exercise program 3 times/week. FFM was assessed by air displacement plethysmography.

**Results:**

At baseline, mean (±SD) BMI was 32 ± 4 kg/m^2^. During intervention, protein intake was 1.13 ± 0.35 g/kg in the high protein groups vs. 0.98 ± 0.29 in the normal protein groups, which reflects a 16.3 ± 5.2 g/d higher protein intake in the high protein groups. Both high protein diet and exercise did not significantly affect change in body weight, FFM and fat mass (FM). No significant protein*exercise interaction effect was observed for FFM. However, within-group analysis showed that high protein in combination with exercise significantly increased FFM (+0.6 ± 1.3 kg, *p* = 0.011).

**Conclusion:**

A high protein diet, though lower than targeted, did not significantly affect changes in FFM during modest weight loss in older overweight and obese adults. There was no significant interaction between the high protein diet and resistance exercise for change in FFM. However, only the group with the combined intervention of high protein diet and resistance exercise significantly increased in FFM.

**Trial registration:**

Dutch Trial Register, number NTR4556, date 05-01-2014.

## Background

Older adults represent the fastest growing population in Europe, but also in the rest of the world [1]. The prevalence of obesity among this age group is 20–30% which has dramatically increased in the past decades [1]. Obesity in older adults is a serious health problem related with multiple chronic health conditions and plays an important role in non-fatal disability [2], which in turn may contribute to lower quality of life [2].

Weight loss leads to metabolic and functional benefits [3]. However, a potential drawback of weight loss in older adults is the accompanying loss of skeletal muscle mass [4], which in turn might accelerate the development of sarcopenia [5]. Strategies to reduce the loss of skeletal muscle mass during weight loss include resistance exercise and sufficient intake of high quality protein [6, 7]. Resistance exercise stimulates muscle protein synthesis, which in turn supports muscle mass preservation and muscle function [8]. In addition, high dietary protein intake has been shown to stimulate muscle protein synthesis in older adults [1, 9–11]. Several studies indicate that, in contrast to young adults, older adults might be resistant to anabolic stimuli from protein, which implies a blunted post prandial response [12, 13].

The number of weight loss trials in overweight or obese older adults is limited, and trials combining resistance exercise with a high protein diet are scarce [14]. We previously studied the effect of a high whey protein-, leucine- and vitamin D-enriched supplement on muscle mass preservation during a 13-week weight loss program including 3 times/week resistance exercise in obese older adults [15]. Subjects in the intervention group received a supplement containing 21 g whey protein (10 servings/wk), whereas the control group received an isocaloric control supplement. This study showed that the intervention group significantly preserved their muscle mass compared to the control group with an effect size of 0.95 kg (95% CI: 0.09;1.81).

Generally, dieticians give dietary advice regarding weight loss treatment based on regular foods, not including any specific supplements. Porter-Starr et al. [16] recently evaluated the effect of a high protein hypocaloric diet using meal-based protein foods in obese older adults over a 6-month period. They found a positive effect on physical performance, but no significant effect on fat free mass (FFM). No studies so far have evaluated the effects of a high protein diet using regular foods with or without resistance exercise on the preservation of FFM during weight loss in older overweight and obese subjects. In the present study we therefore evaluated the effects of a high protein diet and/or resistance training on preservation of FFM, fat mass (FM) loss, waist circumference loss and improvement of handgrip strength and physical performance during a 10-week weight loss trial in overweight and obese adults aged 55 years and over.

## Methods

### Subjects

Overweight and obese men and women (≥55 y) with BMI ≥ 28 kg/m^2^, or BMI > 25 kg/m^2^ with waist circumference > 88 cm (women) or > 102 cm (men), were recruited from the Amsterdam area through local flyers and advertisements. Potential subjects were excluded when they had participated in any weight loss program three months prior to screening; when participation in the resistance training program was considered unsafe according to a physiotherapist; or when they were not able to comply with the full study protocol. All women were postmenopausal and did not use hormone replacement therapy. A full description of the eligibility criteria is online available in the Dutch Trial Register (NTR4556, www.trialregister.nl). The study was approved by the Medical Ethics Committee Independent Review Board Nijmegen, Netherlands (NL43226.072.14) and written informed consent was obtained from all subjects. The study took place from May 2014 through December 2014 at the Amsterdam University of Applied Sciences in The Netherlands.

### Design and randomization procedures

We performed a 10-week randomized controlled trial with a 2-by-2 factorial design combining the factors ‘high protein diet’ and ‘resistance exercise’. Eligible subjects were randomly allocated to either the control group (C) receiving a hypocaloric normal protein dietary advice, the high protein diet group (Pr) receiving a hypocaloric high protein dietary advice, the exercise group (Ex) receiving a hypocaloric normal protein dietary advice with an exercise program, or to the high protein diet and exercise group (PrEx) receiving both a hypocaloric high protein dietary advice and an exercise program. Randomization envelopes with four different codes stratified by gender were generated using a random number generator by the study coordinator. Body composition, waist circumference, handgrip strength and physical performance were assessed at study baseline and after 5 and 10 weeks of intervention.

### Hypocaloric diet and protein advice

All subjects followed a hypocaloric diet of 600 kcal below estimated energy needs [17]. Energy needs were estimated by multiplying measured resting energy expenditure using indirect calorimetry (Vmax Encore n29; Viasys Healthcare, Houten, the Netherlands) with the estimated physical activity level using a 3-day physical activity record. Prescribed dietary protein intake was 0.8 g/kg body weight (BW) for the normal protein dietary advice, and 1.3 g/kg for the high protein dietary advice (using current BW for BMI < 30 kg/m^2^ or using BW at BMI 27.5 kg/m^2^ for those with a BMI ≥ 30 kg/m^2^) [18].

For each subject the amount of energy (kcal) and protein (g) was calculated and incorporated in the dietary advice, which was given at study baseline, together with a specific food variation list for either the high protein or the normal protein diet. Foods were not provided. During intervention, subjects of all groups received five dietary consultations; two times during a face-to-face visit at week 5 and 9, and three times by telephone in week 2, 4 and 7. Dietary intake was assessed by a 3-day food record at baseline, after 5 and 10 weeks of intervention. Intake after 5 and 10 weeks was used to evaluate compliance to the prescribed diet. Food records were checked for completeness during study visits and additional information about unclear items or amounts was obtained. Total energy and macronutrient intakes were calculated using a computerized Dutch Food Composition Table [19].

### Exercise program

The exercise program involved resistance training 3 days a week for 1-h sessions. The training started with a 10-min warming up followed by two sets of 50 s of the following exercises: squats, lunges, chest press, shoulder press, biceps curls, triceps extensions, standing rows, step-ups and crunches. During the 10-week period the number of sets was gradually increased from 2 – 3 set for all exercises, the time to perform the exercises increased from 50 – 75 s, and resistance was increased by using dumbbells, elastic bands, medicine balls and a step bench. The training ended with 5-min cooling down. The exercise program was developed by certified trainers and a physiotherapist and training sessions were supervised by certified trainers. Attendance to the training sessions was recorded by the trainer.

### Measurement of body composition, waist circumference, handgrip strength and physical performance

Body composition including FFM (primary outcome) and FM was determined using air displacement plethysmography (BODPOD, Life Measurement Inc., Concord, CA). BW was measured on the calibrated scale as part of the BODPOD system. Waist circumference was measured in a standing position halfway between the anterior superior iliac spine and the lower rib after normal expiration. Handgrip strength was measured with an isometric handgrip dynamometer (JAMAR 5030 J1, Sammons Preston Rolyan, Bollingbrook, CA) while the subject was seated with the elbow flexed at 90°. Three consecutive measures of handgrip strength (kg) at both hands were recorded to the nearest 0.1 kg and the sum of the maximum value of left and right hand was calculated. Physical performance was assessed with a 400-m gait speed test (m/s) [20], a 4-m gait speed test (fastest of 2 repetitions of usual gait speed, (m/s)), and a chair stand test (s) [21].

### Statistical analysis

Double-data entry was performed and discrepancies were checked and adjusted. Statistical analyses were performed with FFM change as primary outcome. A sample size of *n* = 21 per study group, *n* = 84 in total, provided 80% power to detect an absolute difference of 0.5 kg FFM with SD 0.4 kg and P < 0.05 (2-sided) [22, 23].

Subject characteristics and dietary intake at baseline were compared between groups using an independent samples t-test or the Fisher Exact test. Intention-to-treat analysis was performed using last observations carried forward for subjects with missing week 5 and/or week 10 measurements. Between group differences on outcome variables were analysed using a mixed linear model including time, protein (high/normal), exercise (yes/no) as fixed factors, subject as random factor and sex and baseline value of the outcome variable as covariates. For all outcome variables the interaction for protein*exercise was tested. This interaction tested whether the effect in the exercise groups is dependent on whether the subjects received the high or the normal protein diet (and vice versa). Within group changes over 10 weeks were estimated using a paired t-test.

Statistical analyses were performed using SPSS software (version 22.0, IBM). Data in text and tables are expressed as means with SD, unless stated otherwise. Statistical significance was defined as a two-tailed *P* < 0.05.

## Results

### Subjects

**Fig. 1:**
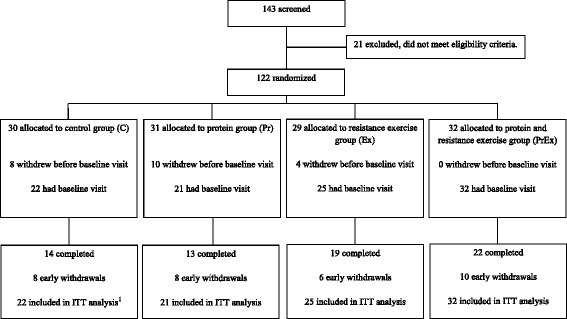
Flow chart of number of subjects screened, randomized, completed intervention and included in the analysis. ^1^ For one subject no body composition data were available at baseline, therefore *n* = 21 subjects were included in the intention to treat (ITT) analysis for the primary outcome fat free mass

We randomized 122 subjects into the four study groups. Before the baseline visits 22 subjects declined study participation for personal reasons. The number of subjects screened, excluded, randomized, and included in the analysis is shown in Fig. 1. Mean age of the study population was 62.4 ± 5.4 y, 36% was male, mean BMI was 32.2 ± 4.3 kg/m^2^ and 66% was obese. There were no relevant differences in subject’s characteristics between the study groups at study baseline (Table 1). Of the 100 subjects with a baseline visit, 32 subjects dropped-out during the study because of adverse events not related to the study (*n* = 7), adverse events related to the study (*n* = 1, lash), personal reasons (*n* = 14), or unknown reasons (*n* = 10).

**Table 1:** Baseline characteristics of 100 obese older subjects by treatment^a^

Characteristic	Control	Protein	Exercise	Protein + Exercise	*p*-value^b^
	(*n* = 22)	(*n* = 21)	(*n* = 25)	(*n* = 32)	
Sex, n (% male)	6 (27%)	8 (38%)	9 (36%)	13 (41%)	0.786
Origin, % Caucasian	82%	86%	68%	84%	0.418
Age, y	63.4 ± 4.3	61.9 ± 6.1	63.1 ± 6.0	61.5 ± 5.1	0.529
Body weight, kg	92.7 ± 5.1	93.0 ± 15.3	90.7 ± 14.7	93.5 ± 14.4	0.912
BMI, kg/m^2^	33.2 ± 4.8	32.1 ± 4.6	32.2 ± 4.7	31.6 ± 3.4	0.584
BMI ≥ 30 kg/m^2^, n (%)	16 (73%)	13 (62%)	16 (64%)	21 (66%)	0.886
Waist circumference, cm^c^	110 ± 13	110 ± 12	107 ± 13	107 ± 9	0.761
Fat mass, %^c^	45.3 ± 8.2	44.7 ± 8.5	43.2 ± 8.7	41.6 ± 7.8	0.383
Fat free mass, kg^d^	51.0 ± 13.1	51.2 ± 10.4	51.5 ± 11.5	54.8 ± 12.7	0.584
Handgrip strength, kg^e^	62.2 ± 22.0	65.2 ± 17.4	70.1 ± 21.8	73.9 ± 24.3	0.234
4-m gait speed, m/s^f^	1.17 ± 0.33	1.32 ± 0.28	1.28 ± 0.22	1.25 ± 0.18	0.284
400-m gait speed, m/s^f^	1.40 ± 0.17	1.42 ± 0.20	1.49 ± 0.21	1.51 ± 0.22	0.133
Time to complete 5 stands, s^f^	13.5 ± 3.2	12.6 ± 3.1	11.1 ± 3.0	11.7 ± 3.4	0.058

^a^Data are presented as means ± SD or as number (percentage); ^b^Significance level (two-sided *p*-value) for comparison between groups using One-Way ANOVA or Chi-square test (sex, origin and BMI group); ^c^n protein + exercise group = 31; ^d^n control group = 21; ^e^Sum of maximum of left and right hand; n protein group = 20; ^f^ n exercise group = 24

### Dietary intake and adherence to exercise program

There were no differences between groups in self-reported mean dietary intake at baseline and the energy reduction during treatment (Table 2). Protein intake during the trial was 1.13 ± 0.35 g/kg/d in the high protein groups. Protein intake was on average 87% of the protein target of 1.3 g/kg/d, with 29% of the subjects reaching this target. In the normal protein groups the protein intake during the trial was 0.98 ± 0.29 g/kg/d, which was on average 123% of the protein target, with 78% of the subjects reaching the protein target of 0.8 g/kg/d. The high protein groups had on average a 16.3 ± 5.2 g/d higher protein intake during intervention (*p* = 0.002) compared to the normal protein groups.

**Table 2:** Dietary intake in the study groups at baseline and during intervention^a^

		Control	Protein	Exercise	Protein + Exercise	*P* value^b^	Protein groups	Control groups	*P* value^c^
		(*n* = 20)	(*n* = 21)	(*n* = 25)	(*n* = 31)		(*n* = 52)	(*n* = 45)	
Energy, kcal/d	Baseline	1928 ± 849	1932 ± 539	1877 ± 522	2061 ± 621	0.730	2009 ± 587	1900 ± 678	0.397
	During intervention	1650 ± 531	1726 ± 449	1569 ± 463	1784 ± 579	0.452	1761 ± 526	1605 ± 490	0.137
Protein, g/day	Baseline	85.7 ± 31.0	82.6 ± 21.4	82.6 ± 23.4	93.2 ± 31.2	0.425	88.9 ± 27.9	83.9 ± 26.7	0.372
	During intervention	76.6 ± 21.1	89.3 ± 22.6	73.9 ± 22.4	92.8 ± 32.9	0.025	91.4 ± 29.0	75.1 ± 21.6	0.002
Protein, g/kg/day	Baseline	0.95 ± 0.36	0.92 ± 0.34	0.93 ± 0.30	1.00 ± 0.31	0.825	0.97 ± 0.32	0.94 ± 0.33	0.662
	During intervention	0.87 ± 0.29	1.02 ± 0.36	0.84 ± 0.23	1.02 ± 0.35	0.081	1.02 ± 0.35	0.86 ± 0.26	0.008
Protein, g/adj_kg/day^d^	Baseline	1.12 ± 0.45	1.04 ± 0.30	1.08 ± 0.36	1.14 ± 0.39	0.820	1.10 ± 0.35	1.10 ± 0.39	0.972
	During intervention	1.00 ± 0.27	1.13 ± 0.33	0.97 ± 0.32	1.13 ± 0.37	0.177	1.13 ± 0.35	0.98 ± 0.29	0.027
Protein, en%^e^	Baseline	18.5 ± 3.3	17.6 ± 3.4	17.9 ± 3.7	18.3 ± 3.6	0.838	18.0 ± 3.5	18.2 ± 3.5	0.814
	During intervention	18.9 ± 2.2	21.1 ± 3.5	18.9 ± 3.0	21.2 ± 4.6	0.033	21.1 ± 4.1	19.0 ± 2.6	0.002
Carbohydrate, en%	Baseline	44.2 ± 5.2	43.3 ± 6.5	45.3 ± 6.0	43.7 ± 8.6	0.760	43.5 ± 7.7	44.8 ± 5.7	0.343
	During intervention	46.2 ± 5.8	40.4 ± 6.1	47.1 ± 6.4	43.3 ± 6.2	0.002	42.2 ± 6.3	46.7 ± 6.1	0.001
Fat, en%	Baseline	33.1 ± 6.3	34.1 ± 5.6	32.3 ± 6.9	32.8 ± 7.7	0.832	33.3 ± 6.9	32.6 ± 6.6	0.625
	During intervention	30.9 ± 5.7	33.3 ± 5.7	29.2 ± 6.9	30.6 ± 7.1	0.214	31.7 ± 6.7	30.0 ± 6.4	0.204

^a^Data represent means ± SD using last observations carried forward; Intake during intervention is mean intake data at week 5 and 10; ^b^Significance level of differences between four groups using One-Way ANOVA; ^c^Significance level of differences between protein and non-protein groups using the t-test; ^d^Protein in g/kg/day with adjusted weight using current weight for BMI < 30 kg/m^2^ or using weight at BMI 27.5 kg/m^2^ for BMI ≥ 30 kg/m^2^, to make it comparable to the protein target; ^e^en% stands for % of energy intake

With respect to the exercise program, mean adherence was 2.8 ± 0.3 times/week.

### Effects on body weight, waist circumference, FFM and FM

**Fig. 2:**
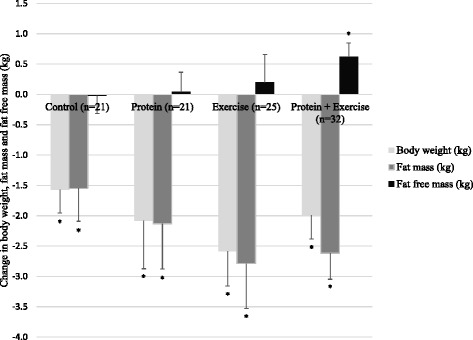
Change in body weight, fat mass and fat free mass in the four study groups. Data represent mean changes over 10 weeks with SEM using last observations carried forward for subjects with missing week 5 and/or week 10 measurements. * indicates within group change using a paired t-test

The 10-week weight loss trial resulted in a significantly decreased BW, waist circumference and FM in all groups. Overall loss in BW was -2.1 ± 2.6 kg, without significant effects of protein and exercise. Comparable results were observed for changes in waist circumference, where a mean decrease of –4 ± 4 cm was observed with no significant effects of protein and exercise (Table 3). Figure 2 shows that the intervention did not significantly affect change in FFM, with exception of the high protein-exercise group which showed a significant increase in FFM of 0.6 ± 1.3 kg (*p* = 0.011). There was no significant effect of high protein and exercise on change in FFM and FM, but exercise significantly decreased body fat percentage with 0.8% (*p* = 0.048). There was no significant protein*exercise interaction for FM and FFM.

**Table 3:** Change in outcome measures at 10 weeks of intervention with protein and exercise effects^a^

	Control	Protein	Exercise	Protein + Exercise	Protein effect		Exercise effect		Protein * Exercise interaction	
	(*n* = 22)	(*n* = 21)	(*n* = 25)	(*n* = 32)	Beta (95% CI)^b^	*P* ^c^	Beta (95% CI)^b^	*P* ^c^	Beta (95% CI)^d^	*P* ^e^
Body weight, kg	−1.7 ± 1.8*	−2.1 ± 3.6*	−2.6 ± 2.9*	−2.0 ± 2.2*	+0.1 (–0.7;1.0)	0.763	−0.3 (−1.1;0.5)	0.472	NS	
BMI, kg/m^2^	−0.6 ± 0.6*	−0.8 ± 1.1*	−1.0 ± 1.0*	−0.9 ± 0.9*	−0.0 (−0.3;0.3)	0.924	−0.2 (−0.5;0.1)	0.213	NS	
Waist circumference, cm	−3 ± 4*	−3 ± 4*	−4 ± 4*	−3 ± 3*^f^	+0.3 (−0.9;1.4)	0.673	−0.4 (−1.6;0.8)	0.555	NS	
Fat mass, kg	−1.5 ± 2.5*^g^	−2.1 ± 3.4*	−2.8 ± 3.7*	−2.6 ± 2.4*	−0.0 (−1.0;0.9)	0.946	−0.8 (−1.7;0.2)	0.124	NS	
Fat percentage, %	−1.0 ± 2.1*^g^	−1.3 ± 2.2*	−1.9 ± 3.3*	−2.1 ± 2.0*	−0.1 (−0.9;0.6)	0.736	−0.8 (−1.6;−0.0)	0.048	NS	
Fat free mass, kg	−0.0 ± 1.4^g^	0.0 ± 1.5	+0.2 ± 2.3	+0.6 ± 1.3*	+0.1 (−0.4;0.7)	0.666	+0.3 (−0.2;0.9)	0.233	NS	
Handgrip strength, kg^h^	+1.8 ± 6.6	−1.7 ± 6.5	−1.8 ± 11.6	+2.0 ± 6.0	−2.2 (−6.4;2.1)	0.311	−1.9 (−6.0;2.1)	0.346	6.2 (0.6;11.8)	0.030
4-m gait speed, m/s	+0.13 ± 0.24*	+0.08 ± 0.26	+0.08 ± 0.13*^i^	+0.20 ± 0.24*	−0.04 (−0.15;0.06)	0.440	−0.04 (−0.14;0.06)	0.476	0.14 (0.00;0.28)	0.045
400-m gait speed, m/s	+0.04 ± 0.15	+0.07 ± 0.10*	+0.07 ± 0.07*^i^	+0.08 ± 0.15*	+0.02 (−0.03;0.06)	0.445	+0.01 (−0.03;0.06)	0.554	NS	
Repeated chair stands, s	−1.6 ± 2.1*	−1.6 ± 1.7*	−1.0 ± 2.7^i^	−1.4 ± 2.7*	−0.1 (−0.9;0.6)	0.703	−0.2 (−0.9;0.6)	0.643	NS	

^a^Data represent means ± SD using last observations carried forward; ^b^Estimate of protein or exercise effect at week 10 using mixed linear model including time, sex, protein (high/normal), exercise (yes/no) and baseline value; ^c^Significance level of estimate of protein or exercise effect at week 10 using mixed linear model; ^d^ Estimate of interaction effect of protein*exercise at week 10 using mixed linear model including time, sex, protein (high/normal), exercise (yes/no), protein*exercise and baseline value only presented when the interaction effect was significant (*P* < 0.1). The effect of the combined protein-exercise intervention can be calculated by summating the beta’s of the protein effect, the exercise effect and the interaction protein*exercise effect; ^e^Significance level of the interaction effect of protein*exercise at week 10 using mixed linear model; ^f^ *n* = 31; ^g^ *n* = 21; ^h^Sum of maximum of left and right hand, n protein group = 20; ^i^ *n* = 24; ^*^ Significant mean change at week 10 within group using a paired t-test; NS not significant (*P* ≥ 0.1)

### Effects on handgrip strength and physical performance

No significant change in handgrip strength was observed over time whereas all physical performance tests improved over time. However, no significant effects of protein and exercise on handgrip strength and physical performance tests were observed (Table 3). There was a significant interaction for protein*exercise for handgrip strength (*p* = 0.030) and 4-m gait speed (*p* = 0.045), indicating that combining a high protein diet with exercise had greater positive effects on handgrip strength and 4-m gait speed than high protein diet or exercise only (Table 3).

## Discussion

In the present randomized controlled trial in overweight and obese older adults during weight loss, we observed no significant effect of the high protein diet (although at a lower level than targeted) and resistance exercise on FFM preservation and no statistically significant interaction between high protein and resistance exercise. However, only in the group with the combined intervention of high protein diet and resistance exercise program, FFM significantly increased.

The recommended dietary allowance (RDA) for protein is 0.8 g/kg/d and is age-independent [24]. However, the recent expert opinion on protein requirements of older adults is higher, and recommended protein intake ranges from 1.0 – 1.2 g/kg/d [25]. Specific recommendations for obese older adults during weight loss do not exist. Weijs et al. [26] showed that protein requirements under the challenged conditions of weight loss may be substantially higher than 0.8 g/kg/d, and are probably even higher than 1.2 g/kg/d in order to preserve muscle mass.

In this study we demonstrated that it is difficult to reach a 1.3 g/kg/d protein intake using a hypocaloric high protein diet based on regular food products (mean intake was 1.13 g/kg/d).

Although subjects in the high protein groups had a 16 g per day higher protein intake compared to the normal protein groups (mean intake was 0.98 g/kg/d), the difference in protein intake might have been too small in order to detect an effect on preservation in FFM. Previously, we studied the effect of a high-whey protein, leucine and vitamin D supplement during weight loss on muscle mass preservation in older obese adults [15]. In that study, the difference in protein intake was 28 g/d with an intake of 1.11 g/kg/d in the intervention group and 0.85 g/kg/d in the control group. This difference resulted in a muscle preserving effect of 0.95 kg. However, besides the difference in protein intake, also other components of the supplement, including leucine, vitamin D and other micronutrients might explain the effect on preservation of FFM in that study.

Two other possible explanations for the absence of a high-protein effect on FFM preservation in the present study should be considered. Firstly, older adults might require a minimum threshold of protein with one eating moment to raise muscle protein synthesis levels. Previous studies showed that a minimal amount of 20 g of high quality protein per meal is needed to stimulate protein synthesis above baseline levels [27]. In our former study, the protein supplement was, ten times per week, supplied as 21 g protein at once [15]. In the current study, only 39% of the subjects in the high protein groups had in total at least one eating moment with ≥ 20 g protein over the recorded days during intervention (week 5 and 10).

A second explanation for the absence of a high-protein effect on FFM preservation is the protein composition of the diet. Whey protein has been shown to be very effective in stimulating postprandial muscle protein accretion in older men [28, 29], which has been ascribed to its fast digestion and to the high leucine content. Since we did not focus on specific types of proteins during dietary counseling it is likely that the amount of leucine known to stimulate muscle protein synthesis (at least 2 g per meal [12]) for older adults was not reached for most subjects in our study.

We observed no overall exercise effect, except for relative fat mass (Table 3). However, when analysing the interaction between gender and exercise a significant interaction for FFM with beta +1.1 kg (95%–CI: –0,0;2,3) was shown, indicating that FFM in males responds stronger to the exercise program than FFM in females. This is in line with expectations based on literature [30].

We observed a significant improvement in physical performance during 10-weeks intervention in all groups. We did, however, not observe an additional improvement in physical performance as a results of higher protein intake or resistance training. A suggested explanation could be that the observed FM loss overruled the possible effects of improvements in physical functioning due to high protein and exercise [31].

Previous studies have shown that on average 25–30% of weight loss is lean mass in older obese adults [14]. In our study, all groups including the control group preserved their FFM. It could be speculated that subjects in the control group increased their level of physical activities and sports activities themselves to compensate for the fact that they were not allowed to participate in the exercise group training sessions. A slight increase in physical activity level during intervention was observed for all groups, and this was not different between the groups, which could partly explain the FFM preservation even in the control group. Another explanation could be the relatively high intake of protein in the control groups (average was 0.98 g/kg), which further reduced the protein intake contrast between groups and might have been beneficial for FFM preservation.

A limitation of this study is the unequally distributed number of subjects that withdrew from participation in the study groups before the baseline measurements. Group allocation could be a reason for declining further participation. Another limitation was the lower than expected magnitude of weight loss, which can be partly explained by the preservation of (C, Pr, Ex groups) or gain (PrEx group) in FFM. Furthermore, we advised a -600 kcal/d reduction in energy intake, which was not achieved based on the analyses of the 3-d food records. Most of previous successful weight loss trials in overweight older adults [14] had weekly group sessions with a dietitian. In our study, the subjects had a bi-weekly consultation, which may also have resulted in the limited weight loss observed. Since the amount of weight loss is modest, the change in FFM is also small. Additionally, the duration of the study might have been too short to achieve sufficient weight loss for group differences in FFM preservation due to protein intake to manifest. Finally, our study was designed and powered to find an effect of protein on FFM with a 0.5 g/kg/d difference between groups; however, only a 0.15 g/kg/d difference in protein intake was achieved, therefore making it difficult to draw firm conclusions regarding a higher versus control protein intake during weight loss with or without resistance exercise.

In conclusion, the lower than targeted protein intake of 1.13 g/kg/d obtained by consuming regular protein rich foods did not significantly affect FFM and FM change during modest weight loss in older overweight and obese subjects. There was no significant interaction between the high protein diet and resistance exercise for FFM. However, only the group with the combined intervention of the high protein diet and the resistance exercise program significantly increased in FFM. This suggests that combining protein with resistance exercise is beneficial for FFM preservation during weight loss in older adults, which should be confirmed by future studies using a larger protein contrast.

## Abbreviations

BW: Body weight
C: Control group
Ex: Exercise group
FFM: Fat free mass
FM: Fat mass
ITT: Intention-to-treat
Pr: High protein diet group
PrEx: High protein diet and exercise group
